# Photodegradation of Bamboo: A Study on Changes in Mechanical Performances

**DOI:** 10.3390/ma16010285

**Published:** 2022-12-28

**Authors:** Silvia Greco, Stefania Manzi, Luisa Molari, Andrea Saccani, Gianfranco Ulian, Giovanni Valdrè

**Affiliations:** 1 Department of Civil, Chemical, Environmental and Materials Engineering, University of Bologna, Via Terracini 28, 40131 Bologna, Italy; 2 Department of Biological, Geological and Environmental Sciences, University of Bologna, Piazza Porta San Donato 1, 40126 Bologna, Italy

**Keywords:** bamboo, photodegradation, UV ageing, *Phyllostachys viridiglaucecsens*, bending test

## Abstract

One of the main concerns in using natural materials in construction, such as bamboo, regards their durability. Ultra violet (UV)irradiation is claimed as a damaging agent; therefore, it is important to study its effect. Several studies have shown that bamboo components such as lignin are subjected to photochemical degradation, but it is not well understood how this affects the mechanical properties of bamboo. The aim of this paper is to explore the correlation between photodegradation and bamboo mechanical performance. Bamboo samples were exposed to accelerated UV ageing for different times (from 6 to 360 h) and then subjected to a four-point bending test. Since one of the ways to stabilize bamboo is to thermally treat it, the tests were conducted on natural untreated bamboo and treated bamboo with a traditional flame treatment. Modifications of the chemical features of the material were analyzed with Fourier Trasform Infra Red (FTIR) spectroscopy, while modifications of the morphological features were analyzed byEnvironmental Scanning Electron Microscopy ESEM and optical microscopy observations. The results show that the bending behavior of bamboo is not compromised by UV exposure up to 360 h. In fact, although a progressive degradation of lignin is reported and cracks in the fiber walls are highlighted from micrographs, no effects were found on the fiber length.

## 1. Introduction

Locally sourced natural materials, such as bamboo, represent a viable alternative for sustainable constructions. Bamboo combines a high mechanical resistance with low specific weight and a very high growth speed thus it provides material and can replace timber in the coming future. One of the main challenges in using natural materials in construction concerns their durability. When exposed to weathering, they undergo changes that can compromise their performance. Evaluation of the mechanical performance of bamboo culm subjected to environmental actions is fundamental to understanding its possible use as a structural material [1]. 

Several studies evaluated changes in the characteristics of bamboo due to temperature, especially because of its potential as a treatment. Zhang et al. [2] studied the effect of exposure at temperatures between 100–220 °C for four duration times (1–4 h) on the physical and mechanical properties of *Phyllostachys pubescens* bamboo. They documented a significant reduction in the content of holocellulose and alpha-cellulose with increasing temperature and duration above 160 °C, which was related to a loss of mass (up to 29%). Moreover, they registered a slight increase of the modulus of elasticity below 200 °C, which then decreased quickly with a decrease of the modulus of rupture after 160 °C.

Changes in bamboo dependent on temperature and the duration of the exposure at a certain temperature were also documented by Nguyen et al. [3] using two Vietnamese bamboo species. In this case, slight changes were estimated at 130 °C, moderate at 180 °C, and high at 220 °C. They also found that the results were influenced by bamboo species and culm provenience. Zhong et al. [4] studied changes in the compressive strength of bamboo scrimbers in a wider range of temperatures (20 to 225 °C). They found that 175 °C was a turning point at which bamboo cellulose and phenolic resin start thermal degradation and compressive strength decreased. Moreover, Lee et al. [5] showed that higher treatment temperatures induced damage to the tissue structures, particularly to the parenchyma cells, bringing larger contact angles on the surface, which ensure favorable hydrophobicity of the bamboo. With FTIR spectroscopy, they also revealed that with increasing treatment temperatures, the intensity of the characteristic absorption peaks of polysaccharides decreased while the intensity of lignin peaks increased. In another study, FTIR and Raman spectroscopies were used to study the effect of smoke treatment [6] on the chemical characteristics of bamboo.

Indeed, there is detailed knowledge regarding the modification properties of bamboo due to thermal exposure, as well as to the damage deriving from the interaction with humidity [7,8], but very few studies have assessed the degradation of lignin when subjected to UV light absorption. Reactions occurring in lignin are considered the main cause of degradation by UV in wood. Alpha-carbonyl, biphenyl, and ring-conjugated double bond structures in lignin can absorb UV light and form chromophores that absorb light, leading to the formation of radical species that are responsible for the wood color changes [9]. Wang et al. [10] analyzed the effect of UV irradiation on changes in lignin in *Phyllostachys pubescens* bamboo species. A relevant decrease in the intensities of the characteristic aromatic lignin peak was accompanied by the formation of new carbonyl groups. Scanning electron microscopy (SEM) imaging also highlighted that the fiber wall was significantly damaged after irradiation, with intense cracks that led to a separation of the secondary cell wall. Parenchyma cells showed slight distortion of the cell wall and cracks on the inside of the cell wall. Yu et al. [11] observed an abrupt decrease in the lignin content during the first 5 days followed by a soft decrease until stabilization after 14 days of exposure to UV radiation. The external surface was covered by degraded products protecting the inner layer; then, after the erosion of degradation products, the surface color turned grey. Another study [12] observed significantly lower photodegradation in bamboo compared to wood, with less carbonyl formation and lignin degradation. This was correlated with the color change, which happened in the first 20 h and then remained constant. Lignin is strongly correlated to strength and stiffness in bamboo culm and its degradation could significantly affect the mechanical performance of bamboo structures. Therefore, it is important to understand the effect of external changes on lignin.

Although several analyses have been conducted on changes in the color of the bamboo surface, it is not well understood how the mechanical properties of bamboo are affected by UV exposure. 

This paper aims to understand the correlation between photodegradation and the mechanical performance of the material, that is presently unknown.

## 2. Materials and Methods

### 2.1. Materials

**Figure 1:**
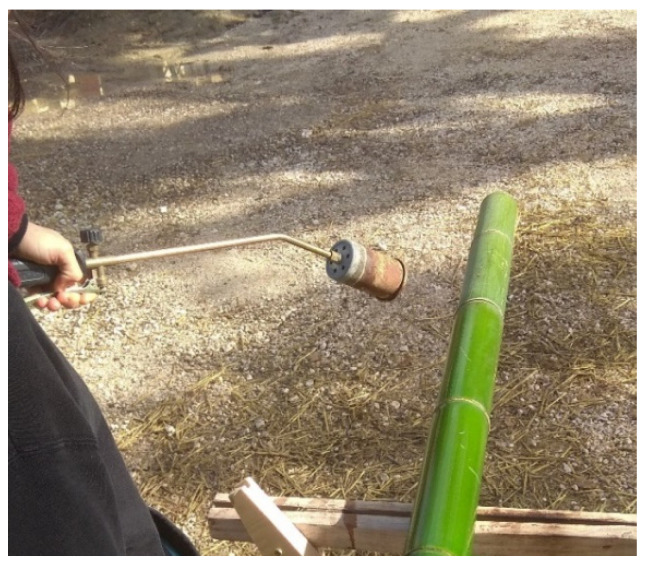
Flame treatment.

**Figure 2:**
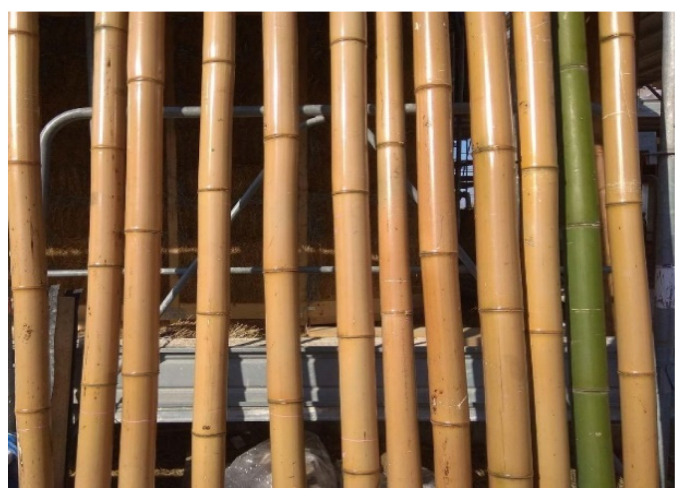
Drying of an untreated green culm and several treated ones.

Three-year-old bamboo culms of *Phyllostachys viridiglaucecsens* species were collected in February 2021 in Macerata (central part of Italy). Storage and sampling have been done according to ISO 22157-1:2019 [13]. The collected bamboo was washed with a pressure washer and then stored vertically in a protected storage room. Two types of bamboo culms have been used in this study. Half of the samples were used in their natural form while half of them were treated with the Abura–Nuki technique, a traditional thermal treatment used in Japan. With the help of a charcoal fire or gas burner, water, oil, and resin are extracted from the culms. A culm with a 50–80 mm diameter and 4–5 m long is subjected to the flame of a portable Liquefied Petroleum Gas (LPG) gas torch (usually used for the application of bituminous membranes) for roughly 5 minutes (Figure 1). During this process, the culm is constantly rotated about its longitudinal axis. The progression of the treatment along the element is controlled by the color change of the skin and the amount of the resin leakage. After about 2 weeks of exposure to the sun, the bamboo becomes shiny, with an agreeable brown tone (Figure 2). The average dimension of the bamboo culm is around 60 mm for the diameter and 5 mm for the thickness.

### 2.2. UV Aging 

**Figure 3:**
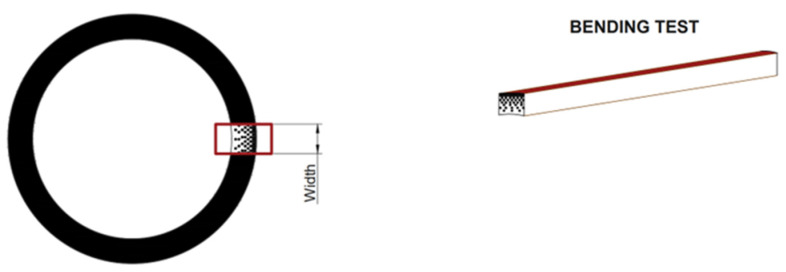
Specimens in the culm for the bending test and microscope observation.

Eleven sticks with straight and defect-free internodes that were a minimum of 220 mm in length were obtained from each culm with a cutter. A screwdriver was used to open the cylinder, trying to respect the fiber path, as shown in Figure 3 (as suggested in UNI 11842:2021 [14] and in [15]). Seven of these sticks coming from the same portion of the culm were exposed to UV rays for different time durations, three of these only to temperature and one was tested untreated for reference. All the specimens were selected from the bottom part of the culms (between 0.5 to 1.5 m from the ground). The cross-section of the specimens is rectangular with one dimension coincident with the thickness of the culm and the other which allows the application of strain gauges.

**Figure 4:**
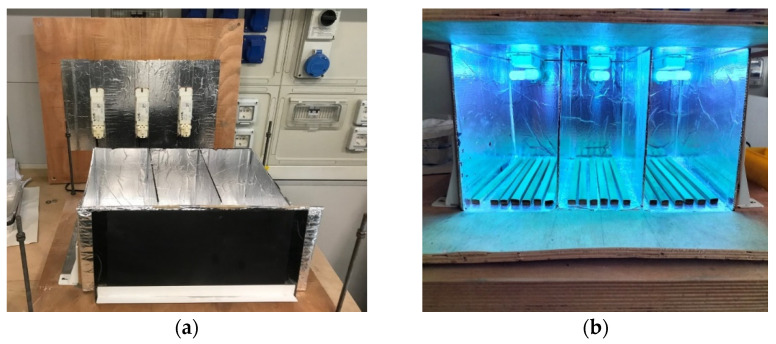
Box used for performing UV irradiation test before (**a**) and during (**b**) irradiation.

The samples were exposed to UV radiation in a box with reflecting walls and UV lamps (Dulux S 9 Watt 78 BLUE UV−A, Osram, Munich, Germany) ). The samples were posed as the external skin was subjected to UV radiation, as happens in real environmental conditions. The specimens were removed after 6, 12, 24, 48, 96, 192, and 360 h. Specimens were posed with the external part exposed to UV rays, as represented in Figure 4. Temperature and relative humidity were measured during the test. The temperature oscillated between 35 and 40 °C during the same day while the humidity was constant around 28 ± 2%. 

To evaluate the specific effect of the temperature on the mechanical behavior, some samples were subjected to a constant temperature treatment of 40 ± 1 °C in a 20 L ventilated oven (Galli, Milano, Italy). The temperature was chosen as the one registered into the box with UV lamps. The time of exposure was 96, 192, and 360 h, and these samples were compared with those exposed to UV rays.

### 2.3. Microscopic Analysis 

To investigate the morphology and possible degradation of the surface, the outer part of the specimens was subjected to attenuated total reflection (ATR) FTIR analysis and to environmental scanning electron microscopy (ESEM) imaging. The attenuated total reflection (ATR) FTIR analysis was performed using a Perkin Elmer Spectrum 3 instrument (Perkin Elmer, Waltham, USA)), fitted with a diamond prism with a resolution of 4 cm^−1^; 64 scans were applied in the measurements. Selected bamboo samples for ESEM analysis were embedded in epoxy resin, then cut and subsequently polished with abrasive pastes of gradually decreasing granulometry to obtain thin sections of 100 µm thickness. Morphological analyses were carried out with a Field Emission Quattro S ESEM (Thermo Fisher, Waltham, USA) ), using the following operation conditions: low vacuum (atmosphere of water) of 170 Pa, electron beam acceleration voltage of 12 kV, with an objective lens aperture of 30 µm. These conditions were selected according to Monte Carlo simulations of the electron beam/specimen interaction, as done in previous works [16]. Low vacuum (LVD) and angular backscatter (ABS) detectors were employed to collect secondary and backscattered electrons, respectively.

### 2.4. Bending Tests

**Figure 5:**
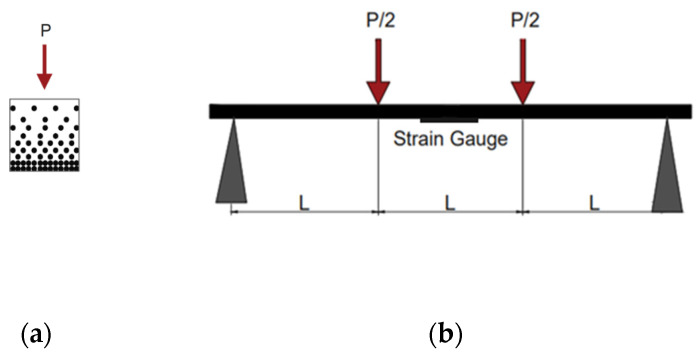
Bending test set-up. (**a**) Cross section of the specimen with the external side of the bamboo culm wall posed in the lower part (tension). (**b**) Four-point bending scheme.

After the exposure, a four-point bending test was carried out on each specimen to evaluate the effect on mechanical properties. In particular, the irradiated surface, which is the external one in the bamboo culm wall, was posed to test the tension, as shown in Figure 5. 

A span length of 60 mm was chosen to allow a span-to-depth ratio of 15. The samples were tested on an Amsler Otto Volpert-Werke GMBH D−6700 test machine (Neu-Ulm, Germany). Bending strength was calculated as:

(1) $$\sigma_{b,ult}=\frac{M_{ult}y}{J}$$

where $\sigma_{b,ult}$ is the bending strength parallel to the fiber, $M_{ult}$ = $P_{ult}$L/6 is the value of the bending moment at the maximum load, *y* is the distance between the external fiber from the neutral axis, and *J* is the moment of inertia of the section. A strain gauge was applied in the lower part of each specimen to measure the deformation of the external fiber stressed in tension. At the end of the test, the humidity was measured for each culm according to ISO 22157:2019.

## 3. Results

### 3.1. Bending Tests

**Figure 6:**
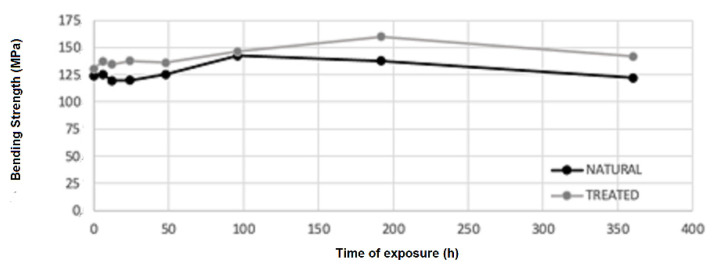
Bending test set-up. Change in bending strength according to different times of exposure for the average values of natural or treated samples.

**Figure 7:**
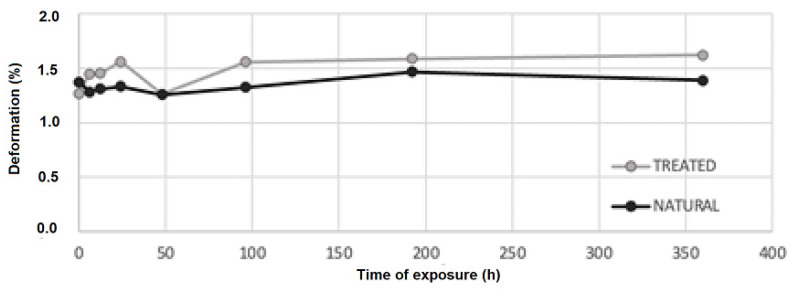
Change in deformation of the external part of the wall fiber according to different times of exposure for the average values of natural and treated samples.

Values of bending strength and deformation are shown in Table 1. Samples seem to not be affected by the irradiation process. An increment in strength occurs after 48 h of irradiation (Figure 6). After 96 h, bending strength shows an increment of 31% concerning the initial one for the average of natural samples, while 12% for the average of treated samples. After 192 h, bending strength shows an increment of 21% concerning the initial one for the average of both types of samples. After this time, strength started decreasing, but remained higher than the initial one. Deformation (see Figure 7) shows a little increase after the irradiation process, but there are no significant changes at different times of exposure. 

Treated samples show values of strength and deformation slightly higher for the average values but are not statistically significant. The same behavior has been noticed for treated and untreated samples without UV exposure in [17]. In that case, the density for treated bamboo samples also showed a slight increment, and this could be a possible reason for this behavior.

**Figure 8:**
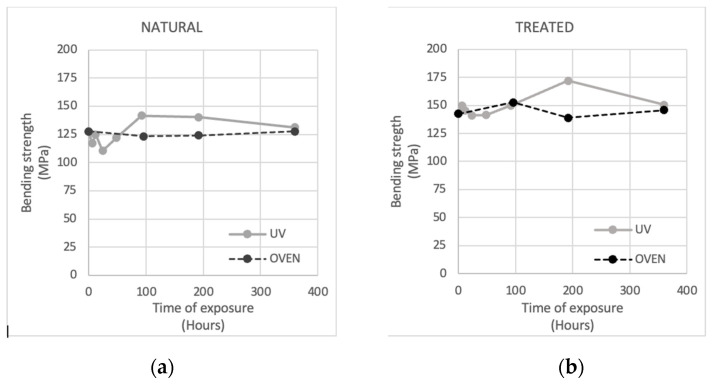
Effect on bending strength of the exposure to the temperature of 40 °C compared to the exposure to UV rays. (**a**) Natural case; (**b**) Treated case.

The effect of temperature was compared with that of UV rays, as in Figure 8 and Table 2. It seems that there is no influence of temperature on increasing the bending strength. Values of bending strength become similar again at 360 h when UV-exposed samples started the downtrend of the strength. 

**Figure 9:**
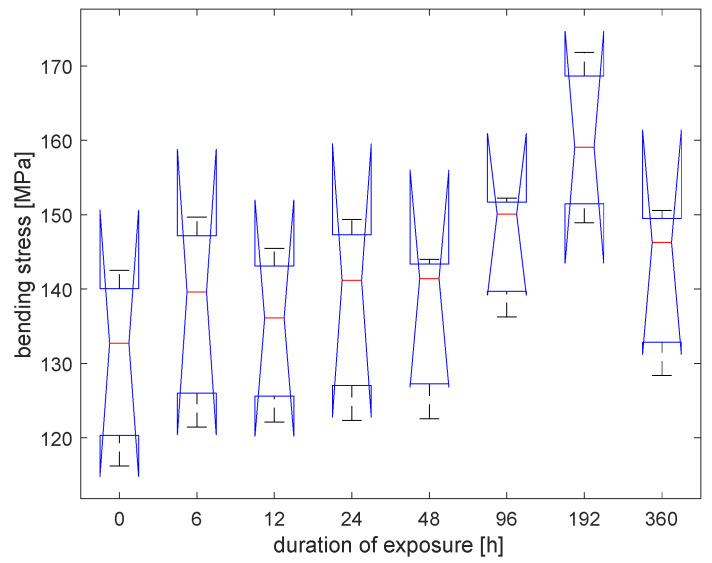
Box plot for the specimens with different durations of the UV irradiation.

**Figure 10:**
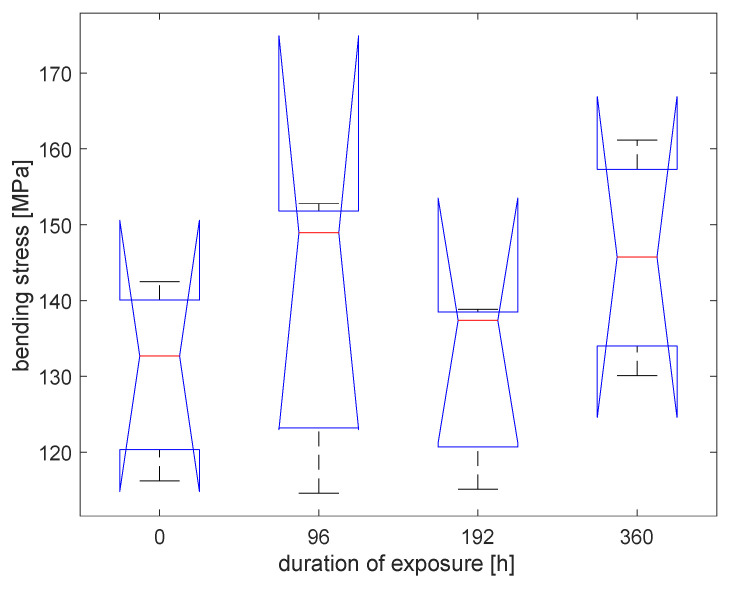
Box plot for the specimens with different durations of exposure to 40 °C.

A statistical analysis has been performed with a one-way ANOVA in Matlab R2021b. Bending strength values are statistically indistinguishable. Figure 9 shows the box plot of the data obtained at different UV exposures. A trend in the average values is visible, even if the groups are not distinguishable considering threshold values for the p-value of 0.05, as reported in Table 3. Figure 10 shows the box plot of the data obtained at different exposures to the temperature of 40 °C. The groups of data are not statistically distinguishable (Table 4 collects the related *p*-values).

### 3.2. IR and Microscopical Analysis 

**Figure 11:**
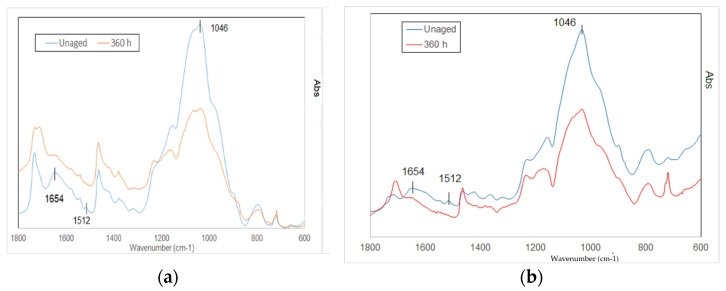
ATR FTIR spectra of unaged and UV (360 h) aged natural (**a**) and treated (**b**) samples.

Figure 11 reports the FTIR results performed on the virgin and 360 h of UV exposure for untreated and treated samples, respectively. Lignin is the most affected component by this type of degradation. As reported in previous studies on bamboo photo-oxidation [12], the progressive degradation of lignin is evidenced by the reduction of two bands, i.e., the ones at 1512 and 1654 cm^−1^. In both Figure 11a and Figure 11b, the intensity of the two highlighted peaks is reduced. In addition, the broad band at 1046 cm^−1^ decreases on account of the UV stress. This peak is also related to the lignin phase [18], and confirms the degrading reactions affecting this phase.

**Figure 12:**
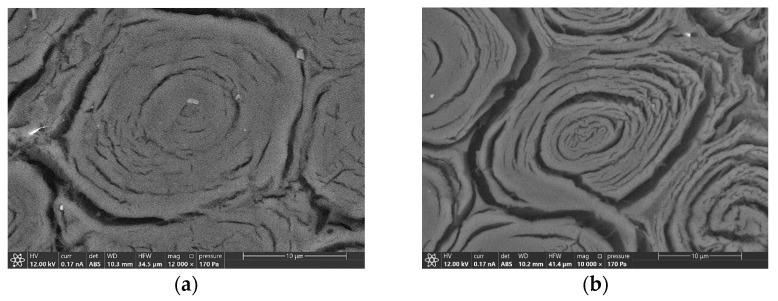
Backscattered electrons ESEM micrograph of a fiber of an unaged sample (**a**) and a UV (192 h) aged specimen (**b**), both taken from the external part of a vascular bundle of the central part of the culm wall.

**Figure 13:**
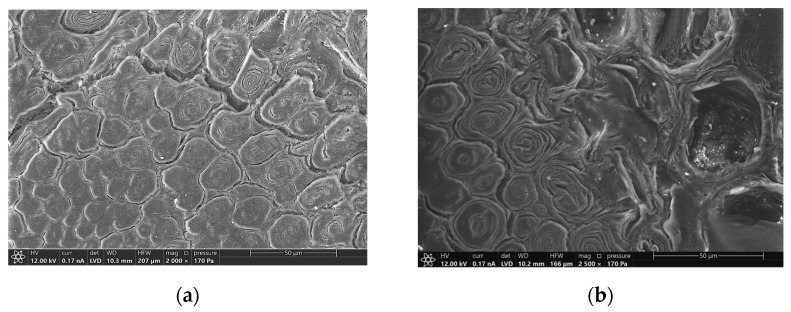
Secondary electrons ESEM images of the border between fibers and ground parenchyma of unaged (**a**) and UV (192 h) aged (**b**) samples, both collected from a vascular bundle of the central part of the culm wall.

**Figure 14:**
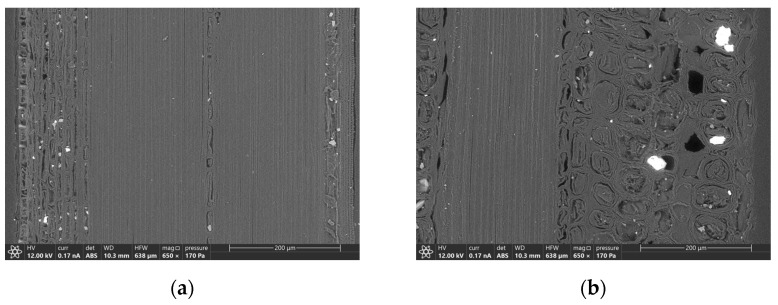
Backscattered electrons ESEM micrograph of the longitudinal section of an aged sample (192 h), taken from the external surface (**a**, on the left) to the inner surface (**b**, on the right) of the culm wall.

Modification of the morphology of fibers was also demonstrated by ESEM investigation and agrees with previous studies [10]. A fiber from a 192-hour aged sample was compared to the one of the virgin samples and are shown in the backscattered electron image of Figure 12. The former presents a visible separation of the layers constituting the fiber wall and some cracks into the same layer. The latter results more compact and only small fissures between vascular bundles and ground parenchyma, or in some cases, between fibers into the same vascular bundle were present. Moreover, fibers of the external part of the vascular bundles are more affected by deterioration in comparison with the inner one (see Figure 13), while no significant differences have been observed regarding the different position of the fibers in the culm wall. From a longitudinal section of the samples, no modification is visible in the fiber structure, as shown in Figure 14. This result could explain the mechanical behavior shown in bending tests: as fibers are undamaged along their longitudinal development, bending performances are not influenced by UV deterioration.

**Figure 15:**
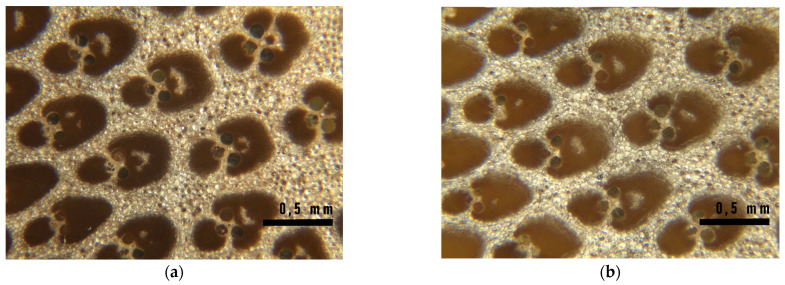
Optical microscopy images showing holes in the fiber island of the vascular bundles of the middle part of the culm wall for natural (**a**, on the left) and treated (**b**, on the right) samples after 192 h of exposure to UV irradiation.

Regarding reflection optical microscopy observation, some parenchyma zones into the fibers island of the vascular bundles appeared into the aged samples, as reported in Figure 15. It is not well understood if it is due to the procedure of preparation of the surface for the observation because of a weakening of the fibers, or to a natural reduction of the area of the fiber island, but it is not present in virgin samples and is therefore a consequence of the UV exposure.

## 4. Conclusions

UV ageing, although claimed as one of the main causes of bamboo damage in all the construction manuals, has only been partially studied previously and not yet from the point of view of the macroscopical mechanical damage. Some studies prove changes in color [12] or chemical components in the surface [11], but they did not focalize on the effects on the mechanical behavior.

This paper aims to investigate the modifications in the mechanical performances of bamboo when exposed to UV radiation. 

Bamboo samples of the species *Phyllostachys viridiglaucecsens* were subjected to irradiation from 6 to 360 h and then to a four-point bending test. This study shows that:

- After 48 h of exposure, bending strength starts growing, reaching an increment of 31% (149 MPa), concerning the initial value (113 MPa), at 96 h of exposure.
- After 96 h, bending strength starts declining slightly from the higher value but remains higher than the initial strength; after 360 h of exposure, it is 8% of the initial one (122 MPa).
- There are no significant changes in deformation at different times of exposure.
- Modifications of the chemical features of the material have been analyzed with FTIR spectroscopy and a progressive degradation of lignin is reported.
- Modifications of the morphological features have been analyzed by ESEM and optical microscopy observations, and cracks in the fiber walls are highlighted from micrographs, as reported in [10]. No effects have been found on the fiber length.

Two types of material have been used: natural and treated with flame bamboo. Treated samples exhibit similar behavior, although the strength of the specimen after treatment is higher:

- After 48 h of exposure, bending strength starts growing, reaching an increment of 23% (160 MPa) concerning the initial value (130 MPa) at 192 h of exposure.
- After 192 h, bending strength starts declining slightly from the higher value but remains higher than the initial strength, as it is around the 8% after 360 h of exposure (142 MPa).
- There are no significant changes in deformation at different times of exposure.
- Modifications of the chemical features of the material have been analyzed with FTIR spectroscopy and a progressive degradation of lignin is reported for the virgin samples.

The modification of bending performance cannot be considered due to the influence of temperature, as verified throughout a comparison between the bending performance of samples exposed to temperature modification at 40 °C and UV rays. 

In conclusion, from the experiments performed, it can be assumed that UV exposure up to 360 h causes a decrease in the content of lignin and a change in the surface, but do not compromise the structure of the fibers along their longitudinal development. This is an important result that can allow the use of bamboo along the culm axis, which is actually the main way for this material to be used.

This study can be implemented in the future to further study the effect of UV radiation over a longer period.

## Figures and Tables

**Table 1:** Average values and standard deviation (in brackets) for bending strength and deformation of the external part of bamboo thickness, referring to treated and natural samples exposed to different UV radiation hours.

Hours of Exposure	Natural Samples		Treated Samples	
	*σ_b,ult_* [MPa]	*ε* [%]	*σ_b,ult_* [MPa]	ε [%]
**0**	113.26 (18.94)	0.98 (0.16)	130.48 (13.30)	1.27 (0.17)
**6**	125.12 (11.44)	1.27 (0.02)	136.92 (14.31)	1.45 (0.09)
**12**	119.45 (15.14)	1.23 (0.1)	134.55 (11.76)	1.45 (0.17)
**24**	119.75 (14.09)	1.36 (0.04)	137.62 (13.86)	1.56 (0.13)
**48**	125.30 (10.46)	1.3 (0.05)	135.99 (11.71)	1.26 (0.2)
**96**	149.72 (12.55)	1.2 (0.17)	146.18 (8.67)	1.56 (0.14)
**192**	137.69 (17.33)	1.51 (0.13)	159.95 (11.49)	1.59 (0.09)
**360**	122.20 (26.70)	1.39 (0.04)	141.74 (11.78)	1.62 (0.13)

**Table 2:** Difference in bending strength between samples exposed to UV rays and those exposed to temperature for three different lengths of exposure.

Hours of Exposure	*σ_b,ult_* [MPa] (St Dev)			
	UV Rays		Oven	
	Natural	Treated	Natural	Treated
**96**	149.72 (12.55)	146.18 (8.67)	123.25 (9.03)	138.00 (21.03)
**192**	137.69 (17.33)	159.95 (11.49)	120.62 (20.30)	130.45 (13.31)
**360**	122.20 (26.70)	141.74 (11.78)	132.10 (6.16)	145.67 (15.54)

**Table 3:** *p*-values of the different groups of duration of UV irradiation.

	6 h	12 h	24 h	48 h	96 h	192 h	360 h
0 h	0.997	0.999	0.995	0.999	0.759	0.125	0.941
6 h	−	1.000	1.000	1.000	0.978	0.346	0.999
12 h		−	1.000	1.000	0.931	0.245	0.995
24 h			−	1.000	0.986	0.381	0.999
48 h				−	0.964	0.304	0.999
96 h					−	0.853	0.999
192 h						−	0.614

**Table 4:** *p*-values of the different groups of exposure to the temperature of 40 °C.

	96 h	192 h	360 h
**0 h**	0.919	1.000	0.668
**96 h**		0.918	0.951
**192 h**			0.667

## Data Availability

The data which are not directly available on the paper can be required to the authors.
